# *Plasmodium falciparum* Drug Resistance Genes *pfmdr1* and *pfcrt In Vivo* Co-Expression During Artemether-Lumefantrine Therapy

**DOI:** 10.3389/fphar.2022.868723

**Published:** 2022-05-24

**Authors:** M. Silva, M. Malmberg, S. D. Otienoburu, A. Björkman, B. Ngasala, A. Mårtensson, J. P. Gil, M. I. Veiga

**Affiliations:** ^1^ Life and Health Sciences Research Institute (ICVS), School of Medicine, University of Minho, Braga, Portugal; ^2^ ICVS/3B’s–PT Government Associate Laboratory, Braga, Portugal; ^3^ SLU Global Bioinformatics Centre, Department of Animal Breeding and Genetics, Swedish University of Agricultural Sciences, Uppsala, Sweden; ^4^ Section of Virology, Department of Biomedical Sciences and Veterinary Public Health, Swedish University of Agricultural Sciences, Uppsala, Sweden; ^5^ College of STEM, Johnson C. Smith University, Charlotte, NC, United States; ^6^ Department of Microbiology, Tumor and Cell Biology, Karolinska Institutet, Stockholm, Sweden; ^7^ Muhimbili University of Health and Allied Sciences, Dar Es Salaam, Tanzania; ^8^ Department of Women’s and Children’s Health, International Maternal and Child Health (IMCH), Uppsala University, Uppsala, Sweden; ^9^ Center for Biodiversity, Functional & Integrative Genomics, Faculdade de Ciências, Universidade de Lisboa, Lisboa, Portugal; ^10^ Global Health and Tropical Medicine, Institute of Hygiene and Tropical Medicine, Nova University of Lisbon, Lisbon, Portugal

**Keywords:** malaria, mRNA, *in vivo*, *P. falciparum*, artemether-lumefantrine

## Abstract

**Background:** Artemisinin-based combination therapies (ACTs) are the global mainstay treatment of uncomplicated *Plasmodium falciparum* infections. *Pf*MDR1 and *Pf*CRT are two transmembrane transporters, associated with sensitivity to several antimalarials, found in the parasite food vacuole. Herein, we explore if their relatedness extends to overlapping patterns of gene transcriptional activity before and during ACT administration.

**Methods:** In a clinical trial performed in Tanzania, we explored the *pfmdr1* and *pfcrt* transcription levels from 48 patients with uncomplicated *P. falciparum* malaria infections who underwent treatment with artemether-lumefantrine (AL). Samples analyzed were collected before treatment initiation and during the first 24 h of treatment. The frequency of *Pf*MDR1 N86Y and *Pf*CRT K76T was determined through PCR-RFLP or direct amplicon sequencing. Gene expression was analyzed by real-time quantitative PCR*.*

**Results:** A wide range of pre-treatment expression levels was observed for both genes, approximately 10-fold for *pfcrt* and 50-fold for *pfmdr1.* In addition, a significant positive correlation demonstrates *pfmdr1* and *pfcrt* co-expression. After AL treatment initiation, *pfmdr1* and *pfcrt* maintained the positive co-expression correlation, with mild downregulation throughout the 24 h post-treatment. Additionally, a trend was observed for *Pf*MDR1 N86 alleles and higher expression before treatment initiation.

**Conclusion:** *pfmdr1* and *pfcrt* showed significant co-expression patterns *in vivo*, which were generally maintained during ACT treatment. This observation points to relevant related roles in the normal parasite physiology, which seem essential to be maintained when the parasite is exposed to drug stress. In addition, keeping the simultaneous expression of both transporters might be advantageous for responding to the drug action.

## Introduction

*Plasmodium falciparum* malaria is responsible for nearly half a million deaths every year, despite the recent worldwide reduction in the incidence of the disease. Worryingly, the trend of decreasing malaria mortality has stagnated since 2015, followed recently by a steep increase due to the impact of the Covid-19 pandemic (WHO, 2021). This later tendency highlights the necessity to develop new strategies to tackle the disease, which in turn requires new valuable knowledge on parasite biology.

*Plasmodium falciparum* is notorious for its high capacity to develop drug resistance. Since the first reports of quinine resistance, more than 100 years ago (Rodrigues Coura, 1987), this parasite has demonstrated the ability to circumvent the action of essentially every antimalarial treatment deployed to a significant scale. Artemisinin combination therapies (ACTs) were introduced to curb the phenomenon of drug resistance, being the mainstay in uncomplicated malaria therapy since their global adoption during the first decade of the 21st century. Nevertheless, even for these generally highly efficacious therapies, reports suggest a progressive trend of reduction in efficacy. This has been first recognized for artesunate-mefloquine (Price et al., 2004) and artesunate-amodiaquine (Holmgren et al., 2006; Humphreys et al., 2007). In addition, the recent failure of dihydroartemisinin-piperaquine (DHA-PPQ) therapy in Cambodia just a few years after its formal implementation in the country, showcases the remarkable resilience of this parasite to drug pressure (Amaratunga et al., 2016; Amato et al., 2017; Hamilton et al., 2019). Nevertheless, and until recently, the globally most used ACT, artemether-lumefantrine (AL) has shown remarkable resilience.

In Africa, the global malaria epicenter, AL is the key ACT, present in essentially every national malaria program of the continent and represents the backbone of malaria clinical management. Accordingly, recent reports of decreased AL efficacy have been met with considerable concern (Plucinski et al., 2015; Plucinski et al., 2017).

The main culprit of artemisinin reduced susceptibility, clinically characterized as an infection with a clearance half-time of >5 h (Dondorp et al., 2009), has been identified as the *P. falciparum* Kelch 13 (*Pf*K13) (Straimer et al., 2015), with other players possibly augmenting the susceptibility (Veiga et al., 2014). However, therapy failure has been more likely to result from failure of the long half-life partner drug. As so, it is of importance to monitor and find novel markers of partner drug resistance. At present, most partner drugs are from the quinoline family and while a complete understanding of the molecular basis of resistance is still not clarified, two transporters, *P. falciparum* multidrug resistance protein 1 (*PfMDR1*) and *P. falciparum* chloroquine resistance transporter (*PfCRT*), have been shown to be pivotal in the parasite response to ACT partner drugs.

*Pf*MDR1 (also referred to as the P-glycoprotein homologue, Pgh) is a ca. 1419 amino acid protein comprising 12 trans-membrane domains, and a member of the ATP-binding cassette (ABC) superfamily (Foote et al., 1989). *Pf*MDR1 is oriented toward the digestive vacuole (DV) lumen and works as an importer of solutes toward this organelle, including antimalarials (Rohrbach et al., 2006). *Pf*MDR1 single nucleotide polymorphisms (SNPs), and of particular importance, the N86Y allele, have been associated with *in vivo* and *in vitro* sensitivity to a range of quinoline antimalarials (e.g., mefloquine, lumefantrine, amodiaquine) and artemisinin derivatives (Veiga et al., 2016; Gil and Krishna, 2017).

*Pf*CRT was identified as the main determinant of chloroquine resistance (Fidock et al., 2000). *Pf*CRT is a 424 amino acid protein with 10 transmembrane domains, a member of the drug-metabolite transport superfamily (Martin and Kirk, 2004), and localizes in the DV membrane of the parasite being able to pump antimalarial drugs out of this organelle (Martin et al., 2009). The *Pf*CRT physiological function has been proposed to be related to the export of host-derived peptides to the cytoplasm (Shafik et al., 2020). The critical chloroquine resistance mutation K76T and other polymorphisms have been documented to influence parasite sensitivity to quinolines (Sidhu et al., 2002; Johnson et al., 2004; Sisowath et al., 2009; Agrawal et al., 2017) and artemisinin compounds (Sidhu et al., 2002; Cooper et al., 2005).

Increased gene expression has been associated with drug resistance in *Plasmodium falciparum* by the causal link of augmented protein levels and treatment outcome. This is evidenced by *pfmdr1* duplications leading to mefloquine and lumefantrine decreased efficacy (Price et al., 2006; Calcada et al., 2020), and through the association of *plasmepsin 2* and *3* amplifications with the collapse of piperaquine containing ACTs in Southeast Asia (Silva et al., 2020). From this background, it is conceivable that changes in transcription activity can also play a role in therapy response. Accordingly, we have previously shown that the levels of *pfk13* expression negatively correlate with *P. falciparum* infection clearance time (Silva et al., 2019) upon AL treatment. In this study, we sought to evaluate the *in vivo* gene expression of the two most-known modulators of drug resistance, *pfcrt* and *pfmdr1*, during AL treatment.

## Materials and Methods

### Clinical Material—Study Site and Sample Collection

The trial was conducted at Fukayosi Primary Health Care Centre, Bagamoyo District, Tanzania (Carlsson et al., 2011) (Clinical Trials (United States), identifier NCT00336375) between March and May of 2006. This was when AL had not yet been nationally implemented in the country, allowing the analysis of an ACT naive population still not genetically or epigenetically modulated by the selective pressure of these drugs.

Briefly, 50 patients between 1 and 10 years old, 31 girls and 19 boys, were included with microscopically confirmed acute uncomplicated *P. falciparum* malaria defined as a 2 –200 *P. falciparum*/µL of blood, an axillary temperature of ≥37.5°C, and whose parent or legal guardian gave their written informed consent. Exclusion criteria included anemia (hemoglobin <70 g/L), significant malnutrition, signs of severe malaria, or any other danger signs.

All patients were hospitalized and followed up for 72 h during the AL treatment course. Six weight-adjusted doses of Coartem® (20 mg of artemether plus 120 mg of lumefantrine, Novartis AG, Basel) were administered under supervision at 0, 8, 24, 36, 48, and 60 h. Patients weighing 5–14 kg received one tablet/dose, patients weighing 15–24 kg received two tablets/dose, and patients weighing 25–34 kg received three tablets/dose. A clinical assessment was performed at 0, 2, 4, 8, 16, 24, 36, 48, 60, and 72 h. Parasite densities were determined in Giemsa-stained and parasite clearance (PC) was evaluated as a proportion of patients with positive microscopy (Carlsson et al., 2011). Venous blood samples (1 ml) were obtained at all clinical assessment points. The total amount of blood harvested for each patient during the study period was inside the general recommended parameters of <5% of the patient’s blood volume (Howie, 2011). As expected for uncomplicated malaria patients, the majority of the circulating parasites were in the ring and early trophozoite forms. For molecular analysis, 500 μL of collected blood was mixed with 500 μL of 2x Nucleic Acid Purification Lysis Solution (Applied Biosystems, Fresno, CA, United States) and stored under liquid nitrogen conditions. Upon arrival to the laboratory, the materials were archived at -80°C until RNA extraction.

The study was compliant with the ethical principles of the Declaration of Helsinki and ethically cleared by the National Institute for Medical Research, Dar-es-Salam, Tanzania, and the Regional Ethics Committee, Stockholm, Sweden, for the downstream molecular analysis of bio-samples.

### RNA Extraction and mRNA Analysis

RNA was extracted using an ABIPRISM H6100 Nucleic Acid PrepStation (Applied Biosystems, Fresno, CA, United States), with the total RNA quality and quantity measured with an Agilent RNA 6000 Pico total RNA assay in an Agilent 2100 Bioanalyser™ (Agilent, Santa Clara, CA, United States). cDNA synthesis was performed with a High-Capacity cDNA Reverse Transcription Kit (Applied Biosystems, Fresno, CA, United States) for the first six time points (T0, T2, T4, T8, T16, and T24 h). Two out of 50 patient sample sets were excluded from this study due to low total RNA quality.

Real-time analyses of the *pfcrt* and *pfmdr1* transcripts were performed with an ABIPRISM® 7900HT Sequence Detection System (Applied Biosystems, Fresno, CA, United States). TaqMan® probes and primer sequences for target genes, *pfmdr1* (PF3D7_0523000), *pfcrt* (PF3D7_0709000), and the endogenous control gene seryl-tRNA synthetase (PF3D7_0717700) that was shown to be transcribed stably throughout different intraerythrocytic stages (Bozdech et al., 2003; Veiga et al., 2010; Magallon-Tejada et al., 2016; Ngwa et al., 2017). Oligonucleotide primers and conditions are well established, having been previously developed for *in vitro* gene expression investigations with culture adapted clones (Veiga et al., 2010), *pfmdr1*, (6-FAM, TAMRA probe 5′-GTA​TTT​AAT​AAC​CCT​GAT​CGA​AAT​GGA​ACC​TTT​G-3′, and primers 5′-TGC​ATC​TAT​AAA​ACG​ATC​AGA​CAA​A-3′ and 5′- TCG​TGT​GTT​CCA​TGT​GAC​TGT-3′); *pfcrt*, (6-FAM, MGB probe 5′-CTA​TAT​CCA​TGT​TAG​ATG​CC-3′, and primers 5′-CGA​CAC​CGA​AGC​TTT​AAT​TTA​CAA​T-3′ and 5′-AAG​ACC​TAT​GAA​GGC​CAA​AAT​GAC-3′); seryl-tRNA synthetase (VIC, TAMRA Probe 5′-TGA​AAC​TAT​AGA​ATC​AAA​AAG​GTT​ACC​ACT​CAA​ATA​CGC​T-3′ and primers 5′-CCT​CAG​AAC​AAC​CAT​TAT​GTG​CTT-3′ and 5′-TGT​GCC​CCT​GCT​TCT​TTT​CTA-3′). Supplementary Figure S1 details the probes’ efficiency and further details can be resourced from the original report (Veiga et al., 2010). Amplification reactions were done in triplicate in 384-well plates with 10 µL total volume reactions containing TaqMan® Gene Expression Mastermix (Applied Biosystems, Fresno, CA, United States), 300 nM of each forward and reverse primer, 100 nM of TaqMan® probe, and 4 µL of cDNA. The thermal cycle program was 50°C for 2 min, 95°C for 10 min, and forty cycles of 95°C for 15 s and 60°C for 1 min.

### *pfcrt* and *pfmdr1* Molecular Genotyping

*Pf*CRT K76T and *Pf*MDR1 N86Y, Y184F and D1246Y alleles were analyzed for all samples before treatment initiation. *Pf*CRT K76T and *Pf*MDR1 N86Y were further analyzed during the treatment period for the time points 2 h (T2), 4 h (T4), 8 h (T8), 16 h (T16), and 24 h (T24) hours.

PfCRT K76T SNP was evaluated through published PCR-RFLP methods (Veiga et al., 2006). *Pf*MDR1 N86Y, Y184F, and D1246Y SNPs were analyzed through direct PCR amplicon sequencing, as previously described (Malmberg et al., 2013). The *pfmdr1* gene copy number was performed in all samples using housekeeping gene tubulin beta chain putative (PF3D7_1008700) with probes and primers, as described by Price et al. (2004). *P. falciparum* DNA from 3D7 and FCB reference strains were used as the calibrator and the positive control, respectively (known copy number variation). All reactions were performed in an ABI PRISMH 7000 Sequence Detection System (Applied Biosystems™, Fresno, CA, United States).

### Data Analysis

RealTime StatMiner® software (Integromics, http://www.integromics.com/StatMiner) was used to obtain the ΔC_t_ (target transporter gene C_t_ values minus endogenous PF3D7_0717700 control gene C_t_ values) for each patient. The amplification efficiency was used as the correction factor as previously defined (Veiga et al., 2010). Formula 2^-ΔCt^ was used to obtain the fold change expression pre-treatment (T0). For each patient, the *pfmdr1* and *pfcrt* transcript fold change relative expression was calculated by the 2^-ΔΔCt^ method (Livak and Schmittgen, 2001), in which the ΔΔC_t_ was calculated using the ΔC_t_ from T0 as the calibrator of the matching patient sample. The Shapiro–Wilk test was used for assessing the data set normality and Spearman correlation applied to assess linear relations between *pfmdr1* and *pfcrt* expression variation throughout time.

Patients’ data and *pfmdr1* and *pfcrt* transcript expressions were stratified into two groups defined as containing the wild type or mutant allele for *Pf*MDR1 N86Y and *Pf*CRT K76T SNP (mixed infections excluded (Table 1). The Mann–Whitney test was used on the patient data and *pfmdr1* and *pfcrt* relative expression data.

**TABLE 1:** *pfmdr1* and *pfcrt* expression data.

	PfMDR1				PfCRT			
	**Total (N=48)**	N86 (n=10)*	Y86 (n=25)*	**P value**	**Total (N=48)**	K76 (n=23)*	T76 (n=15)*	**P value**
	**Mean ± SD (min - max)**	**Mean ± SD (min - max)**	**Mean ± SD (min - max)**		**Mean ± SD (min - max)**	**Mean ± SD (min - max)**	**Mean ± SD (min - max)**	
Expression (2–∆Ct)								
0 h	12.84 ± 11.53 (2.19 - 56.79)	19.68 ± 17.43 (4.96 - 56.79)	11.34 ± 10.20 (3.76-17.39)	0.10	4.71 ± 2.44 (1.45 - 13.80)	4.92 ± 2.26 (3.64 - 6.92)	3.85 ± 1.92 (1.45 - 5.83)	0.55
Relative expression (2–∆∆Ct)								
2 h	1.05 ± 0.72 (0.30 - 3.43)	0.79 ± 0.29 (0.3 - 1.15)	1.25 ± 0.90 (0.58 - 2.23)	0.39	0.90 ± 0.47 (0.28 - 2.91)	0.80 ± 0.20 (0.6 - 1.05)	1.10 ± 0.75 (0.28 - 2.91)	0.89
4 h	0.96 ± 0.85 (0.18 - 3.62)	0.55 ± 0.25 (0.18-1.01)	1.14 ± 1.03 (0.55 - 2.02)	0.08	0.77 ± 0.32 (0.25 - 1.99)	0.73 ± 0.18 (0.49 - 1.2)	0.92 ± 0.47 (0.57 - 1.99)	0.66
8 h	0.92 ± 1.00 (0.12 - 4.66)	0.50 ± 0.28 (0.12-1.08)	1.11 ± 1.18 (0.37 - 1.96)	0.12	0.63 ± 0.49 (0.09 - 2.80)	0.54 ± 0.21 (0.37 - 0.79)	0.83 ± 0.77 (0.39 - 2.8)	0.44
16 h	0.76 ± 0.83 (0.09 - 3.65)	0.37 ± 0.20 (0.11-0.77)	0.90 ± 0.98 (0.28 - 1.23)	0.06	0.37 ± 0.32 (0.05 - 1.89)	0.35 ± 0.17 (0.25 - 0.79)	0.42 ± 0.47 (0.18 - 1.89)	0.92
24 h	0.70 ± 0.79 (0.03 - 3.30)	0.35 ± 0.20 (0.09-0.74)	0.83 ± 0.91 (0.03-1.26)	0.08	0.37 ± 0.30 (0.06 - 2.01)	0.34 ± 0.15 (0.13 - 0.52)	0.37 ± 0.25 (0.15 - 0.99)	0.77

*Mixed infections excluded from statistical calculations.

## Results

### Baseline *pfcrt* and *pfcrt* mRNA Levels

**FIGURE 1:**
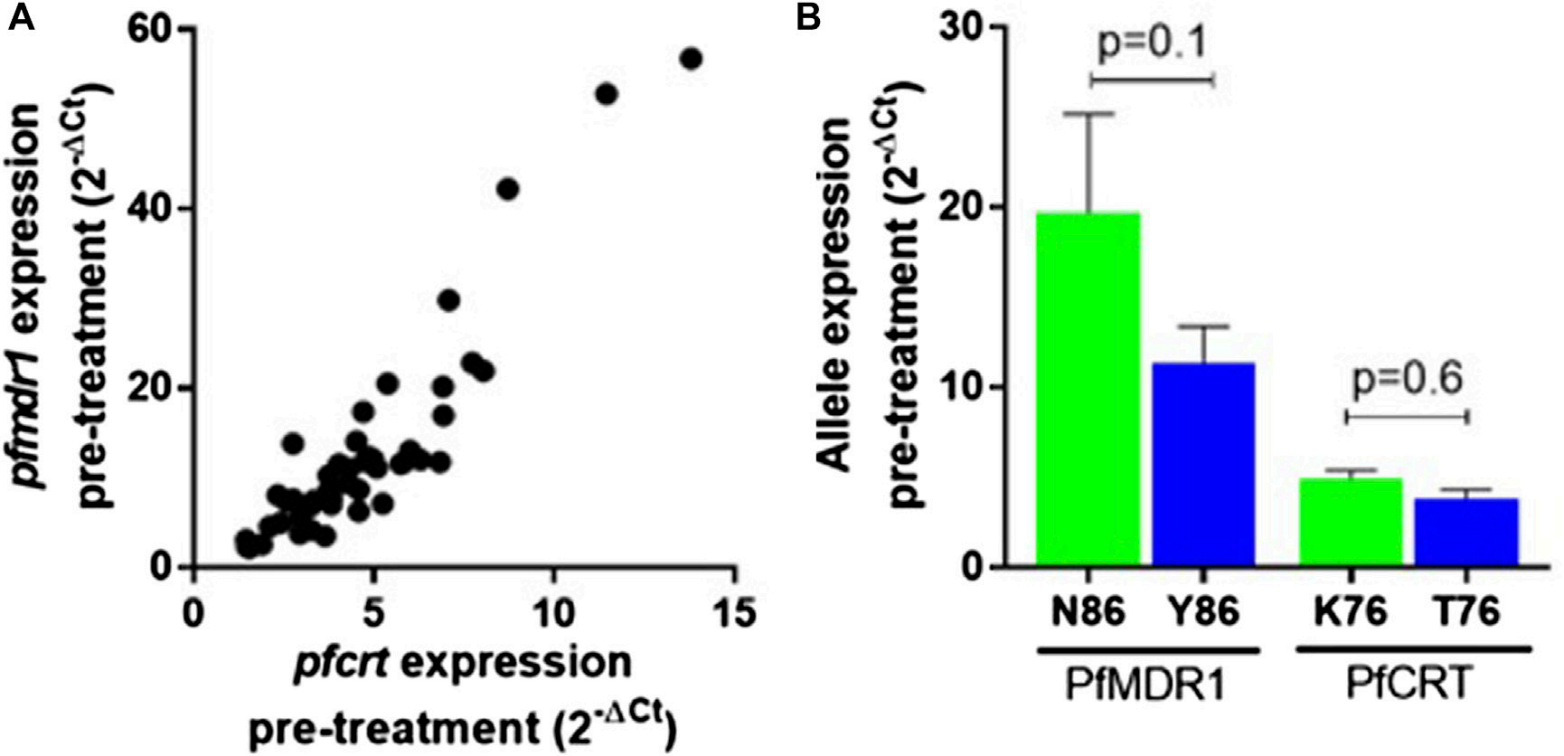
*pfmdr1* and *pfcrt* expression in the infection before treatment with artemether-lumefantrine. **(A)** Correlation between the *pfmdr1* and *pfcrt* mRNA levels (Spearman correlation, R = 0.85, *p* < 0.0001). Expression fold change was calculated by the 2^–∆Ct^ method normalized with the housekeeping gene seryl-tRNA synthetase. **(B)** Mean ± SEM expression according to the *Pf*MDR1 N86Y and *Pf*CRT K76T SNP status. Statistical evaluations comparing allele variants were performed using the two-tailed Mann–Whitney U-test. Mixed infections were excluded from the analysis.

*Pfmdr1* and *pfcrt* transcript data obtained from 48 out of the 50 infections were analyzed at the six time points T0, T2, T4, T8, T16, and T24. Blood sampling at T0, T8, and T24 was done just before AL administration. A large range of mRNA baseline values (T0, pre-treatment) was observed for both genes. *pfcrt* transcripts span from 1.45 to 13.80-fold as compared with the internal control gene (PF3D7_0717700), whereas *pfmdr1* showed a larger window from 2.19 to 56.79-fold (Table 1). The most striking observation was the strong positive correlation of the large range of mRNA baseline values between *pfcrt* and *pfmdr1* (Spearman, R=0.82) supporting the possibility of these genes being functionally associated with the normal physiology of the parasite (Figure 1A).

### Post-Artemether-Lumefantrine Treatment *pfmdr1* and *pfcrt* mRNA Levels

**FIGURE 2:**
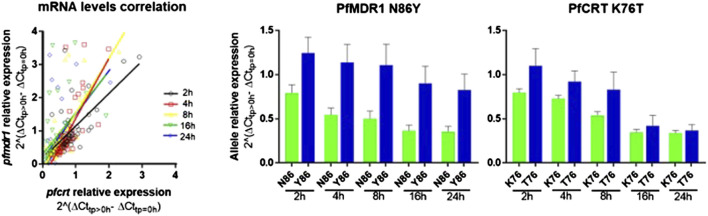
*pfmdr1* and *pfcrt* expression in patients’ post-treatment with artemether-lumefantrine. **(A)** Figure on the let: Correlation between the *pfmdr1* and *pfcrt* mRNA levels (Spearman correlation, T2: R = 0.55, *p* < 0.0001; T4: R = 0.79, *p* < 0.0001; T8: R = 0.67, *p* < 0.0001; T16: R = 0.60, *p* < 0.0001; and T24: R = 0.61, *p* < 0.0001). Post-treatment transcripts were measured from 2 h up to 24 h. The average expression fold change was calculated by the 2^–∆∆Ct^ method normalized with the housekeeping gene seryl-tRNA synthetase and calibrated with paired infection data before treatment initiation (T0). **(B)** Bar plot of *pfmdr1* and *pfcrt* allele relative expression to T0 of the 48 infections discriminating by allele and time point, data presented as mean ± SEM.

After AL treatment initiation, *pfmdr1* and *pfcrt* expressions were analyzed relative to the expression before treatment of the matching patient. An intra-infection positive correlation between *pfcrt* and *pfmdr1* responses was observed for all six monitored time points after treatment (Figure 2A).

The most frequent individual occurrence for both genes was a decrease in the *pfmdr1* and *pfcrt* mRNA levels during the first 24 h on treatment initiation. The *pfmdr1* relative expression decreased from 1.05 ± 0.72-fold (±SD) in T2 to 0.70 ± 0.79-fold (±SD) in T24, decreasing at all time points. The *pfcrt* relative expression decreased from 0.90 ± 0.42-fold (±SD) in T2 to 0.37 ± 0.30-fold (±SD) in T24, decreasing at all time points, except from T16 to T24 which remained constant. Of note, a subset of infections (17%, 8/48) showed a clear rise in *pfmdr1* mRNA levels during the treatment period (threshold ≥1.5 fold).

All enrolled patients were tested for infections carrying the *pfmdr1* increased copy number, with no such events having been detected.

### *Pf*MDR1 and *Pf*CRT Alleles and mRNA Levels

The *Pf*MDR1 N86Y and *Pf*CRT K76T mutations are considered risk factors for AL treatment failure (Sisowath et al., 2005; Sisowath et al., 2009). A pertinent question is if these mutations are associated with allele-specific upregulation of the respective genes. Comparing the abundance of baseline transcripts, we observed a pattern for higher mRNA transcripts when parasites contained the wild-type allele (Figure 1B), with *Pf*MDR1 N86 having a fold change of 19.7 ± 17.4 (±SD) versus the Y86 11.3 ± 10.2 fold change and *Pf*CRT K76 having a fold change of 4.9 ± 0.5 *vs* the T76 3.8 ± 0.5 (Table 1). These patterns anyway did not reach statistical significance for both genes.

Concerning post-treatment expression levels, we did not detect significant differences between the *Pf*MDR1 N86 and Y86 or *Pf*CRT K76 and T76 alleles carrying parasites, at any time point (Figure 2B; Table 1). The only observable trend was that the *Pf*MDR1 86Y allele had a less steep decrease of expression over the course of treatment from 1.25 ± 0.90-fold (±SD) on T2 to 0.83 ± 0.91-fold (±SD) on T24 compared with *Pf*MDR1 N86 allele, which decreased from 0.79 ± 0.29-fold (±SD) on T2 to 0.35 ± 0.20-fold (±SD) on T24.

The *Pf*CRT K76 and T76 alleles decreased in relative expression during the treatment from 0.80 ± 0.20-fold (±SD) and 1.10 ± 0.75-fold (±SD) on T0 to 0.34 ± 0.15-fold (±SD) and 0.37 ± 0.25-fold (±SD) on T24, respectively. T2 was the only time point at which a *Pf*CRT allele (T76) had an increase in the mean of relative expression compared with pre-treatment.

Parasite treatment response, measured by parasite clearance (PC) time did not differ between *Pf*MDR1 N86Y and *Pf*CRT K76T SNP distribution (Table 2). PC_50_ for *Pf*MDR1 N86 was 6.48 ± 2.57 h (±SD) compared with 86Y of 6.09 ± 3.31 h (±SD). PC_50_ for *Pf*CRT K76 was 5.67 ± 2.46 h (±SD) compared with 76T of 5.69 ± 3.63 h (±SD).

**TABLE 2:** Characteristics of the studied population and differential PfMDR1 N86Y and PfCRT K76T SNP distribution.

	Total (N=48)	PfMDR1 N86 (n=10)*	PfMDR1 Y86 (n=25)*	P value	PfCRT K76 (n=23)*	PfCRT T76 (n=15)*	P value
	Mean ± SD (min - max)	Mean ± SD	Mean ± SD		Mean ± SD	Mean ± SD	
Age (months)	49.88 ± 29.68 (12 - 119)	45.00 ± 23.19	50.52 ± 33.00	0.89	58.22 ± 32.54	35.47 ± 22.97	0.04
Weight (kg)	14.24 ± 5.46 (8 - 30)	12.70 ± 3.34	14.15 ± 6.12	0.92	16.12 ± 6.49	11.53 ± 2.83	0.03
Parasitemia ^#^	60.51 ± 51.30 (2.12 – 200.4)	79.14 ± 60.51	65.04 ± 54.32	0.66	68.89 ± 58.25	56.61 ± 51.87	0.91
Hemoglobin	100.50 ± 16.88 (71 - 134)	103.6 ± 19.75	97.64 ± 14.34	0.33	103.90 ± 19.51	91.87 ± 12.24	0.07
Temperature (°C)	37.91 ± 0.97 (36.20 - 40.80)	38.51 ± 1.21	37.70 ± 0.97	0.06	38.03 ± 1.14	37.53 ± 0.57	0.37
Slope half-life	2.49 ± 1.12 (0.54 - 5.21)	2.36 ± 1.46	2.64 ± 1.10	0.48	2.50 ± 1.13	2.41 ± 1.34	0.97
PC50 (h)	6.00 ± 3.13 (0.80 - 14.78)	6.48 ± 2.57	6.09 ± 3.31	0.62	5.67 ± 2.46	5.69 ± 3.63	0.94
PC90 (h)	11.60 ± 4.79 (2.07 - 21.80)	11.95 ± 4.63	11.87 ± 5.01	0.87	11.47 ± 4.06	10.73 ± 6.03	0.80
PC95 (h)	14.09 ± 5.68 (2.61 - 26.18)	14.31 ± 5.91	14.52 ± 5.84	0.87	13.96 ± 5.02	13.13 ± 7.13	0.87
PC99 (h)	19.87 ± 7.96 (3.86 - 37.84)	19.79 ± 9.08	20.65 ± 8.02	0.58	19.76 ± 7.44	18.72 ± 9.91	0.91

*Mixed infections excluded from statistical calculations.

^#^Pf/mm^3^ of blood

## Discussion

Protein levels associated with increased gene expression have been related to several drug resistant-phenotypes in *P. falciparum* (Price et al., 2004; Sidhu et al., 2006; Sharma et al., 2013; Amato et al., 2017; Inoue et al., 2018; Calcada et al., 2020).

*Pf*MDR1 expression has been implicated in the resistance to multiple drugs, mostly because of an increased gene copy number. Of note, increased *Pf*MDR1 expression is associated with mefloquine resistance and can lead to the inefficacy of the artesunate-mefloquine combination (Price et al., 2004). Gene expression can modulate protein levels and is a potential mechanism to modulate drug response. There are very few studies assessing the patterns of *P. falciparum* gene expression on ACT in uncomplicated malaria patients. Of interest, albeit artemisinin resistance is mainly associated with decreased *Pf*K13 levels resultant of SNPs (Birnbaum et al., 2020), lower *pfk13* transcript levels *in vivo* have also been linked with longer parasite clearance times, along the course of AL treatment (Silva et al., 2019). In this work, we focused on two key players of parasite resistance against the long half-life quinoline partner drugs, the *pfmdr1* and *pfcrt*.

A large range of baseline expression levels was observed for both *pfcrt* and *pfmdr1*, of approximately 10 and 30-fold, respectively. For *pfmdr1*, these *in vivo* data are supportive of previous expression studies using clinical isolates, where a similar range was reported (Legrand et al., 2012). The reasons for these inter-infection differences are unclear. The possibility of a significant influence from the presence of other intra-erythrocytic stages should be relatively small, considering that it involved patients experiencing fever, a status known to drive parasite cycle synchronization (Kwiatkowski, 1989; Engelbrecht and Coetzer, 2013). Moreover, even if there would be an influence of later stages inter-stage, differences in expression *in vitro* are not in the same order of magnitude, compared with those observed in our study (Young et al., 2005; Veiga et al., 2010).

A central observation was the clear correlation between the expression of both *pfcrt* and *pfmdr1*. This was most evident before treatment initiation but extended itself to post AL drug exposure. These data support *in vitro* reports by Adjalley et al. (2015) with individual clones, which further linked these transporters inside a larger set of co-regulated *loci*. Our work shows that this is a regular event during infection, supporting a complementary role of these food vacuole-located transporters in the parasite regular physiology.

Albeit the majority of the studied parasites did not experience an induction on their *pfmdr1* and *pfcrt* genes, our work shows that such events are not apparently uncommon during infections (particularly *pfmdr1*) (Figure 2). These observations raise the issue of how much “hard-wired” the parasite transcription is when considering clinically relevant *in vivo* situations and analyzing a sufficiently large sample. For most *in vitro* studies—including our own (Veiga et al., 2010)—drug exposure has not been reported to drive large transcriptomic changes. Large differences in the baseline gene expression between *P. falciparum* strains have been proposed to be slow and likely driven by epigenetic processes (Batugedara et al., 2017; Arama et al., 2018). Such processes have been, to a certain extent, showcased in the *in vitro* selection of blasticidin-resistant parasites, based on the epigenetic driven suppression of *clag3* gene expression (Sharma et al., 2013; Chan et al., 2020). From this perspective, it is possible that what we are observing *in vivo* are minor sub-populations already expressing *pfmdr1* and *pfcrt* at more than average levels that happen to be selected during drug treatment. Epigenetic-based mechanisms might co-exist with events of promoter-driven gene upregulation. As referred, increases of >2-fold have been reported *in vitro* upon exposure to common antimalarials using clonal populations (Veiga et al., 2010).

The molecular basis of the observed *pfmdr1/pfcrt* co-expression is not known. Of note, both genes harbor common putative xenobiotic response DNA elements in their 5’ promoter regions (up to 5 Kb upstream of the transcription initiation), especially nuclear receptor-recognition sequences (Johnson et al., 2008). Of interest for this discussion, a number of those are common for both *pfcrt* and *pfmdr1,* including BARBIE (barbiturate-inducible element) boxes, retinoic acid receptor (RAR) and retinoic acid receptor half-site (RXR) elements, and progesterone receptor-binding sites (PRE). It is conceivable that, similar to other eukaryotic systems (Penvose et al., 2019), some of these elements might be mechanistically associated with the observed co-regulation pattern between these two genes. In fact, artemisinin derivatives are known agonist ligands of the nuclear pregnane-X-receptor (PXR) and constitutive androstane receptor (CAR) in higher mammals, leading to an increased activity of targeted genes, through the interaction with regulatory sequences as previously mentioned (Burk et al., 2005; Zang et al., 2014). This includes genes coding for P-glycoprotein (Pgp)-type ABC transporters, evolutionary related to the parasite *pfmdr1* product. Moreover, investigations using luciferase expression constructs to probe *pfmdr1* promoter regions have shown these as significantly responsive (Myrick et al., 2003). Furthermore, when applying the classical ADME inducer phenobarbital, five- to sixfold increases in the *Pf*MDR1 protein content were also reported, albeit *Pf*CRT stayed non-responsive, suggesting no significant involvement in our observations of the BARBIE Box (Johnson et al., 2008). Moreover, it is conceivable that the opportunity for *pfmdr1* to be induced might be ruled by the epigenetic status of the proximal 5′ promoter, with favorable conditions only statistically happening in a fraction of the parasite population at any time point. Further studies will be required to investigate this possibility.

An increased copy number of N86 carrying *Pf*MDR1, which leads to significant increases in this gene transcript and associated protein, is a well-documented factor in the *in vivo* and *in vitro* parasite susceptibility to aminoalcohol quinolines and artemisinins (Veiga et al., 2011; Calcada et al., 2020; Silva et al., 2020). Linking this information with the known importance of the *Pf*MDR1 N86 allele modulating AL therapy in Africa (Malmberg et al., 2013; Venkatesan et al., 2014), we initially hypothesized that in a clinical setting, the carriers of this critical allele would tend to have significantly higher baseline levels of expression. Our results do not support this hypothesis, as N86 carrying parasites did not show a significant difference in *pfmdr1* expression compared with the Y86 ones. In fact, if we consider any trend, it is for a higher expression among the 86Y carriers. The consistently observed selection of 86N alleles during AL treatment is more likely to be associated with SNP-driven changes in the structure of the protein, as predicted from *in silico* studies (Ferreira et al., 2011) and not through a link with increased transcriptional activity.

A major limitation of the present study is the participant numbers, a factor related to the outstanding demands of these types of field studies. In addition, the nature of the study limited the availability of biological material for analysis, precluding the consistent use of multiple internal qPCR controls. However, the project’s exploratory nature did not prevent us from providing new insights into *in vivo* gene expression of two key markers of antimalarial resistance.

In conclusion, this work represents, to our knowledge, the first detailed qPCR-based analysis of the parasite *pfcrt* and *pfmdr1* genes during an ACT clinical exposure. By challenging the general view of a rather inert parasite transcriptome, at least concerning the genes under focus, we highlight the need to link valuable *in vitro* data on the parasite drug response mechanisms to the *in vivo* therapy context.

## Data Availability

The original contributions presented in the study are included in the article/Supplementary Materials, further inquiries can be directed to the corresponding author.

## Supplementary Material

Supplementary Figure S1 qPCR analysis of *Plasmodium falciparum* 3D7 reference strain using TaqManR probes. Standard curve analyses data for comparing amplification efficiency and linearity are tabulated.
