# Supplementary figures and images for: Plasmodium falciparum Drug Resistance Genes pfmdr1 and pfcrt In Vivo Co-Expression During Artemether-Lumefantrine Therapy
Source: Front Pharmacol. 2022 May 24;13:868723. doi: 10.3389/fphar.2022.868723 (PMC9171324; doi:10.3389/fphar.2022.868723)

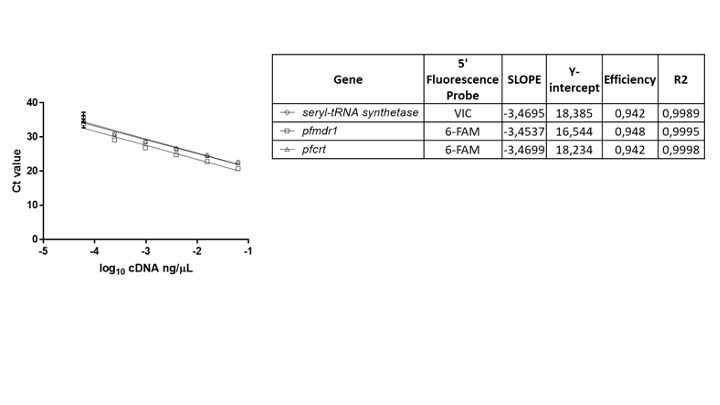

Supplement: Supplementary file 1 [file Image1.jpeg]
